# Bacterial Community Composition Associated with Chironomid Egg Masses

**DOI:** 10.1673/031.012.14901

**Published:** 2012-12-27

**Authors:** Yigal Senderovich, Malka Halpern

**Affiliations:** ^1^ Department of Evolutionary and Environmental Biology, Faculty of Natural Sciences, University of Haifa, Mount Carmel, Haifa 31905, Israel; ^2^ Department of Biology and Environment, Faculty of Natural Sciences, University of Haifa, Oranim, Tivon 36006, Israel

**Keywords:** cloning, DGGE

## Abstract

Chironomids (Diptera: Chironomidae) are the most widely distributed and often the most abundant insect in freshwater. They undergo a complete metamorphosis of four life stages, of which the egg, larva, and pupae are aquatic and the adult is terrestrial. Chironomid egg masses were found to be natural reservoirs of *Vibrio cholerae* and *Aeromonas* species. To expand the knowledge of the endogenous bacterial community associated with chironomid egg masses, denaturing gradient gel electrophoresis and clone analysis of 16S rRNA gene libraries were used in this study. Bacterial community composition associated with chironomid egg masses was found to be stable among different sampling periods. Cloned libraries of egg masses revealed that about 40% of the clones were related to bacteria known to degrade various toxicants. These findings were further supported when bacterial species that showed resistance to different toxic metals were isolated from egg masses and larval samples. Chironomids are found under a wide range of water conditions and are able to survive pollutants. However, little is known about their protective mechanisms under these conditions. Chironomid egg masses are inhabited by a stable endogenous bacterial community, which may potentially play a role in protecting chironomids from toxicants in polluted environments. Further study is needed to support this hypothesis.

## Introduction

Chironomid species (Diptera; Chironomidae), commonly known as “non-biting midges,” are globally distributed and thrive in almost every possible freshwater environment (Armitage et al. 1995). Females lay egg masses containing hundreds of eggs embedded in a thick, gelatinous matrix. Chironomids undergo a complete metamorphosis of four life stages of which the egg, larva, and pupa are aquatic and the adult is terrestrial.

Chironomids have not been known to cause disease or other deleterious effects in humans (Gerardi and Grimm 1982). However, recently, *Chironomus* sp. egg masses have been implicated as a natural reservoir of *Vibrio cholerae* (Broza and Halpern 2001; Halpern et al. 2003, 2004, 2007) and pathogenic *Aeromonas* species (Senderovich et al. 2008; Figueras et al. 2011). Moreover, chironomid adults may disseminate *V. cholerae* and *Aeromonas* between freshwater reservoirs (Broza et al. 2005). Investigation of the bacterial communities that inhabit chironomid egg masses may contribute to understanding chironomids as natural reservoirs of *V. cholerae* and *Aeromonas*. The culturable bacteria that inhabit chironomid egg masses were characterized previously (Halpern et al. 2007), but only the minority of the naturally occurring bacteria can be cultured and characterized on cultivation media. The enrichment media fail to mimic the conditions that all the microorganisms require for proliferation in their natural habitat. Culture-independent methods targeting the 16S ribosomal RNA gene, such as denaturing gradient gel electrophoresis (DGGE) or clone library screening, offer opportunities for the detailed analysis of the structure and species composition of microbial communities.

The aim of this work was to explore and characterize the bacterial community associated with chironomid egg masses using molecular culture independent techniques, namely PCR-DGGE and cloning. Here, it is shown that a stable bacterial community is associated with chironomid egg masses.

## Materials and Methods

### Chironomid sampling

The study was performed in northern Israel near Haifa at the Tivon waste stabilization pond (longitude 699, latitude 618). The egg masses for the DGGE analysis were sampled from May 2005 to September 2005, when chironomids could be easily found. Two egg masses were sampled randomly at each sampling time (except for June 23). The abiotic conditions of the sampling site were described in Senderovich et al. (2008). The egg masses for the clone libraries and for the egg masses and larvae bacterial isolation were sampled at the same pond in September 2007 and August 2008, respectively. Styrofoam boards (25×25 cm) were used as artificial oviposition sites for adult females. Egg masses along the side of the board were collected 24 hours after introducing the board into the water habitat. Larvae were collected from the water. The egg masses and the larvae were brought to the laboratory immediately after collection. Each freshly collected egg mass and larva was treated separately to remove all the bacteria that were not firmly attached by washing and vortexing for 1 minute in sterile saline water. This procedure was repeated five times. The egg masses for the DGGE and cloning analysis were suspended in 2 mL 95% ethanol and kept at 20° C until further examination.

### DNA extraction and chironomid identification

In order to digest the gelatinous matrix of the egg mass and lyse the attached bacteria, each egg mass was treated with 600 µl buffer containing 20 mM Tris-HCl, (pH 8.8 at 25° C), 2 mM EDTA, 1.2% triton, and 10 mg/mL lysosyme at 37° C for 45 min. Afterwards, 30 µl proteinase K (Invisorb Spin Plant Mini Kit, Invitek, http://www.invitek.de/) were added, and the sample was incubated at 65° C for 40 min. The DNA was extracted with Invisorb Spin Plant Mini Kit according to the manufacturer's instructions and stored at -20° C.

The taxonomic identification of chironomid species was performed by PCR amplification and sequencing of *cytochrome oxidase* subunit I gene (Folmer et al. 1994). The sequences were compared to those available in the GenBank databases (http://www.ncbi.nlm.nih.gov) using the standard nucleotide-nucleotide BLAST program (BLASTN, http://www.ncbi.nlm.nih.gov), to ascertain their closest relatives. Sequences were submitted to the GenBank database under accession numbers: JQ025719 and JQ025720.

### PCR amplification for DGGE

The extracted DNA was amplified with puReTaq Ready-To-Go PCR Beads (Amersham Biosciences, Freiburg, Germany) in two successive PCR amplifications. All oligonucleotides used in this study were specific for bacterial 16S rRNA gene. The numbers in the primer names indicate the position of the 5′ nucleotide in the 16S rRNA gene of *Escherichia coli*. A 1501-bp fragment was first PCR amplified using the following primers: HF (GGA TCC AGA CTT TGA TYM TGG CTC AG) (modified from Felske et al. 1997) and 1512R (GTG AAG CTT ACG G(C/T)T AGC TTG TTA CGA CTT) (Felske et al. 1997). The thermal cycling conditions were 94° C for 4 min; 30 cycles at 94° C for 30 sec, at 54° C for 40 sec, and at 72° C for 70 sec; Then at 72° C for 20 min. The amplicons of the first PCR were then used as substrate for the second amplification using 341F GCclamp (Muyzer et al. 1993) and 907R primers (Lane et al. 1985) (566-bp fragment), and that was the substrate for the PCR-DGGE electrophoresis.

### DGGE analysis

For DGGE analysis, the Dcode universal mutation detection system (Bio-Rad, http://www.bio-rad.com/) was used to separate the V3–V5 regions of the PCR products. PCR products were electrophoresed on 6% (wt/vol) Polyacrylamide gels containing a denaturating gradient from 20 to 60% urea and formamide [a 100% denaturant corresponds to 7 M urea and 40% (vol/vol) formamide) in 1xTAE running buffer (2 M Tris base, 1 M glacial acetic acid, 50 mM EDTA]. Migration was performed at 90 V for 16 hr, and the running buffer temperature was kept constant at 60° C. Gel was stained with GelStar Nucleic Acid Stain (0.1 µl/mL) (Cat. No. 50535, Cambrex Bio Science, http://www.cambrex.com/) and photographed by UV GelDoc (Bio-Rad). DGGE bands were excised from the gel, eluted by incubation in 50 µl terile distilled water at 4° C overnight, and amplified. PCR products were sequenced at the Technion Medical School, Haifa, Israel. Amplified fragments on the DGGE were scored as binary data, i.e., presence as 1 and absence as 2, for the homologous bands. TFPGA program version 1.3 (Miller 1997) was used to calculate the Nei (1972/1978) genetic distance.

### Cloning

Analysis of the bacterial community by cloning was performed on two chironomid egg mass samples from the Tivon waste sterilization pond. One sample contained a single egg mass (EM1), and the other sample comprised DNA that was extracted from three different egg masses (EM2) sampled from the same location on the same day. All egg masses were treated as described above to remove all the bacteria that were not firmly attached.

An 896-bp fragment of the 16S rRNA gene was obtained by means of the primers 11F and 907R. The primers and PCR conditions were described above. The purified PCR products were cloned into *E. coli* DH5α using the CR®II-TOPO TA cloning vector (Cat. No. 10351-021, Invitrogen, http://www.invitrogen.com/). About 200 recombinant clones were randomly selected for analysis. The plasmids were extracted using Wizard Plus SV Minipreps kit (Cat. No. A1460, Promega, http://www.promega.com/) and sequenced at the Technion Medical School, Haifa, Israel.

### Clones sequences and DGGE bands analysis

Clone sequences were checked for chimeras using Greengenes database (http://greengenes.lbl.gov/cgi-bin/nphNAST_align.cgi). The remaining sequences were compared with those available in the EzTaxon server (http://www.eztaxon.org/) (Chun et al. 2007) to ascertain their closest relatives. Clone libraries composition was then further analyzed, using the FastGroupII website (http://biome.sdsu.edu/fastgroup/) to obtain the number of the different taxonomic units (OTU's). Sequences with less than 97% similarities in their 16S rRNA gene were considered dissimilar OTU's. Microbial community comparison between the two egg masses' 16S rRNA libraries was performed by the RDP classifier (http://rdp.cme.msu.edu/comparison/comp.jsp). Clone library sequences were submitted under accession numbers JN648150– JN648321. Phylogenetic trees were generated using the neighbor-joining method with NJPlot (MEGA 4.1), based on alignments from CLUSTAL W. The bootstrap values obtained were from 1,000 iterations.

DGGE band's sequences were compared to those available in the GenBank database using the standard nucleotide-nucleotide BLAST program to ascertain their closest relatives. Sequences were submitted to the GenBank database under the following accession numbers: JN630811–JN630815, JN630834– JN630836, JN630818, JN630822, JN630824, JN630832, JN630833, JN630827.

### Isolation of bacteria from the egg masses and the larvae

Freshly collected egg masses and larvae were washed as described above and then crushed in 1 mL saline water by using a sterile glass homogenizer. The homogenate was diluted and aliquots of 0.1 mL from each dilution were spread onto Luria agar (LB) (Himedia) and onto LB agar supplemented with 5 mM of one of the following compounds: K_2_CrOz_4_, Pb(NO_3_)_2_, CuSO_4_·5H_2_O, and ZnCl_2_. Individual colonies with different appearances were picked and streaked on LB agar to obtain single colonies. Isolated colonies were subcultured at least four times before examination. The bacteria were identified by amplifying and sequencing a 1501-bp internal fragment of the 16S rRNA gene using 11F and 1512R primers (Felske et al. 1997), as was described in Senderovich et al. (2008).

The obtained sequences were compared to those available in the EzTaxon server to ascertain their closest relatives. The accession numbers for the sequences are shown in Figure 3.

### Toxicants concentration in the sampled environment

The concentration of heavy metals (lead, chromium, copper, and zinc) at the Tivon waste stabilization pond was determined by Neve Ya'ar extension service laboratory (Ministry of Agriculture) in an Atomic Absorption apparatus (Varian, http://www.varian.com/).

## Results

The taxonomic identification of chironomids that were sampled in the current study was done by using molecular methods. The sampled chironomids were identified as belonging to the species *Chironomus transvaalensis* Kieffer (Diptera: Chironomidae).

### DGGE

**Figure 1.**
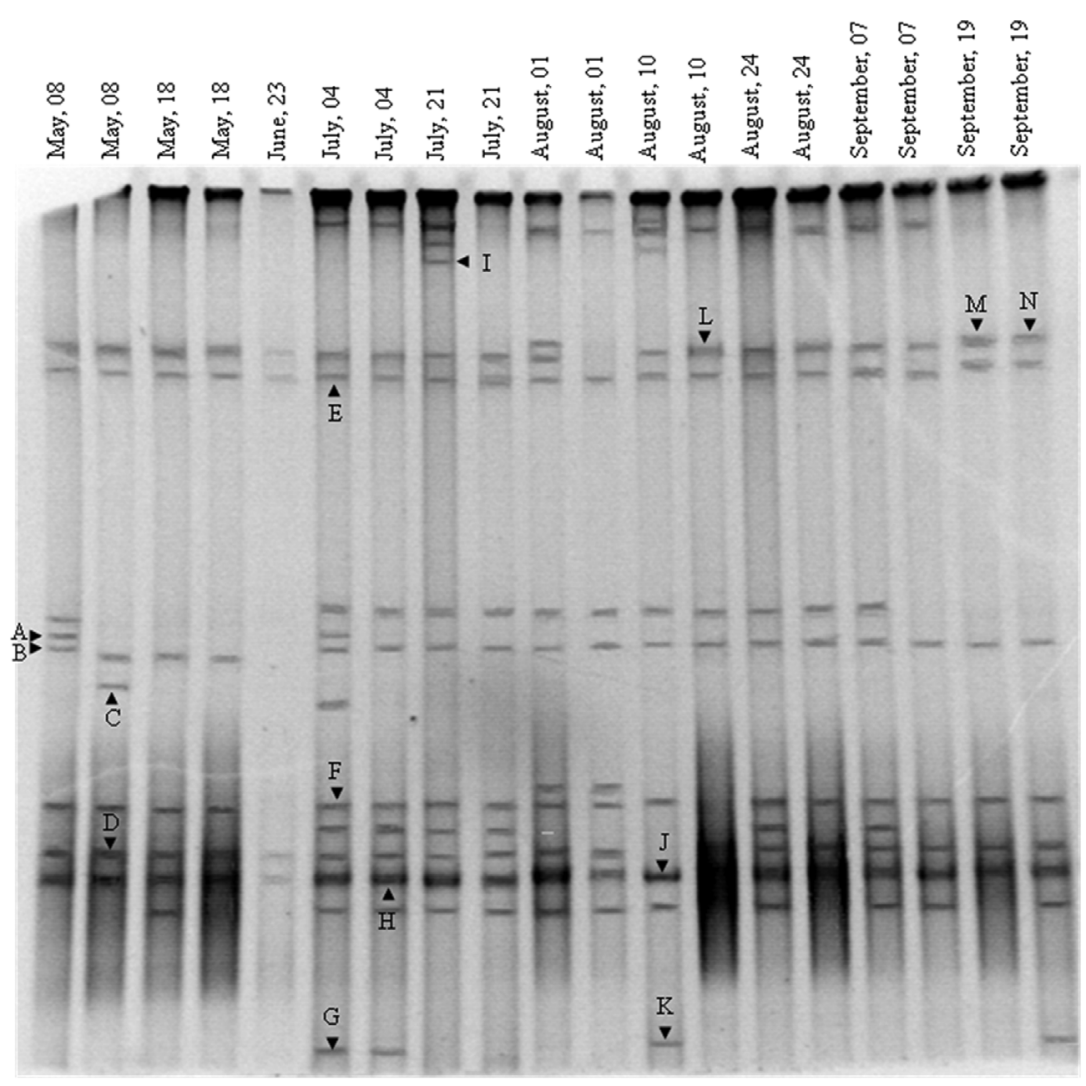
DGGE banding patterns of bacterial community associated with chironomid egg masses. Egg masses (n = 19) were collected between May 2005 and September 2005 at Tivon waste stabilization pond. Partial 16S rRNA gene was PCR-amplified with Bacteria universal primers. Each band in the DGGE gel represents at least one bacterial species. Bands in the same height are considered as belonging to the same species. Fourteen bands (A–N) were excised, sequenced, and compared to those available in the EzTaxon server (http://www.eztaxon.org/) to ascertain their closest relatives. The results of the bands identification (accession numbers): A-E and L-N, *Pseudomonas* spp. (JN630811–JN630815; JN630834–JN630836); F, unidentified *Comamonadaceae* (JN630818); G, *Bacillus* sp. (JN630822)-; H, J, K, *Schlegelella* spp. (JN630824, JN630832, JN630833); 1, *Exiguobacterium* sp. (JN630827). High quality figures are available online.

The PCR-DGGE of the egg masses sampled every other week during a five-month period revealed a unique banding pattern of the bacterial community (Figure 1). The band patterns didn't change significantly between the different sampled egg masses and demonstrated that a stable bacterial community inhabits the egg masses niche (Figure 1). This observation was also supported by the NET s average identity algorithm (1972/1978), which measured 0.79 ± 0.09 SD.

Fourteen bands were excised from the gel in order to determine the DNA sequence and to identify the bacterial species (Figure 1). Eight bands (57%) were identified as *Pseudomonas* sp. and three bands (21%) as *Schlegelella* sp. Other bands were identified as *Bacillus* sp., *Exiguobacterium* sp. and an unidentified *Comamonadaceae* (Figure 1).

### Cloning

**Figure 2.**
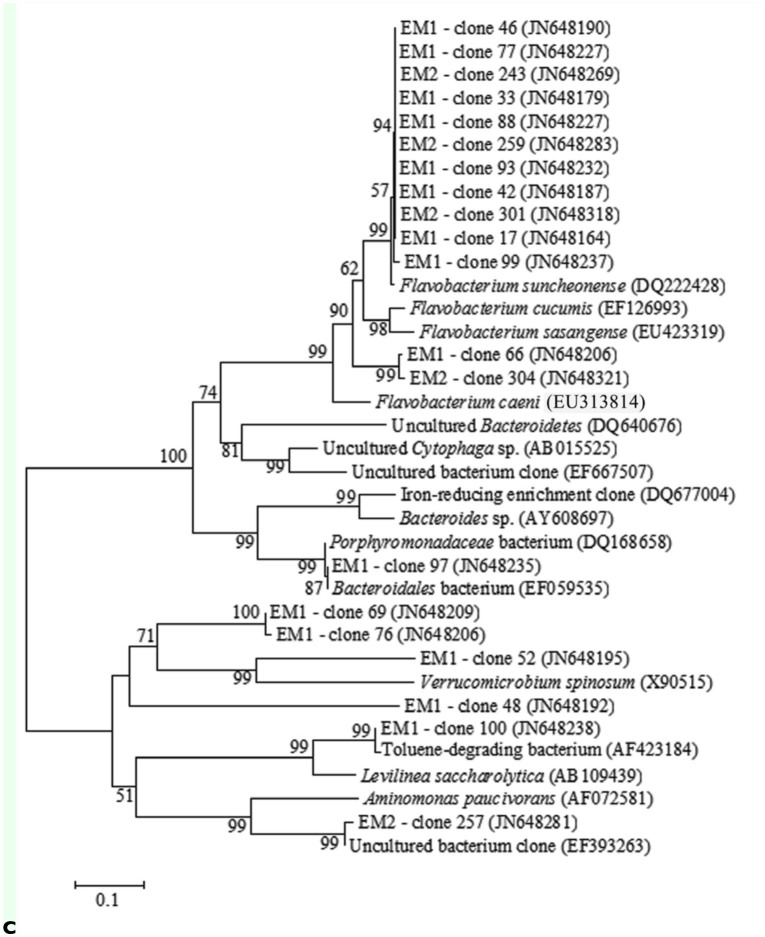
Phylogenetic trees representing selected clones that are closely related to known detoxifying bacteria. (A) *Alphaproteobacteria* and *Gammaproteobacteria* classes. (B) *Betaproteobacteria* class. (C) *Bacteriodetes* and environmental samples. The clones' closest relative sequences were obtained from PubMed database (http://www.ncbi.nlm.nih.gov/pubmed/). Some of these taxons are related to bacterial species that showed the ability to degrade different toxicants such as phenol, benzene, toluene, naphthalene, hexachlorocyclohexane, selenium, aminobenzenesulfonate, (per)chlorate, polychlorinated biphenyl mixture, and polycyclic aromatic hydrocarbons, and detoxify heavy metals. EM1 and EM2 in the isolates name reveal their origin. The sequences alignment was performed using CLUSTAL W program, and the trees were generated using the neighborjoining method with Kimura 2 parameter distances in MEGA 4.1 software. Bootstrap values (from 1,000 replicates) greater than 50% are shown at the branch points. The bar indicates 1–5% sequence divergence. High quality figures are available online.

To obtain additional information on the bacterial community associated with the egg masses, clone libraries were constructed for two egg masses samples. Egg Mass 1 (EMl) contained purified DNA from a single egg mass and Egg Mass 2 (EM2) contained DNA from three egg masses. Most of the clones in both egg mass samples belonged to *Proteobacteria* (32% and 41% in EM1 and EM2, respectively). The *Proteobacteria* phylum was mainly comprised of *Alphaproteobacteria* (6.5% and 8.8%), *Betaproteobacteria* (9.8% and 21.3%), and *Gammaproteobacteria* (13% and 11.3%) classes (in EM1 and EM2, respectively) (Figure 2a, b, c). Another noticeable phylum was *Bacteroidetes* due to *Flavobacteria* class (18.5% and 21% in EM1 and EM2, respectively). Forty percent of the clones in EM1 and 34% in EM2 could not be classified as belonging to a known bacterial phylum.

In total, 169 clones' sequences were obtained after chimeras' removal. Fifty-six and 62 different OTUs were observed in EM1 and EM2 clone libraries, respectively. No significant differences were found between the two egg masses' libraries composition. *Flavobacterium* spp. was the largest OTU and contained 17 clones in EM1's library and 19 clones in EM2's (Table 1, Figure 2). *Pseudomonas* and *Comamonadaceae* identified in the DGGE bands were found in both clone libraries.

**Table 1.**
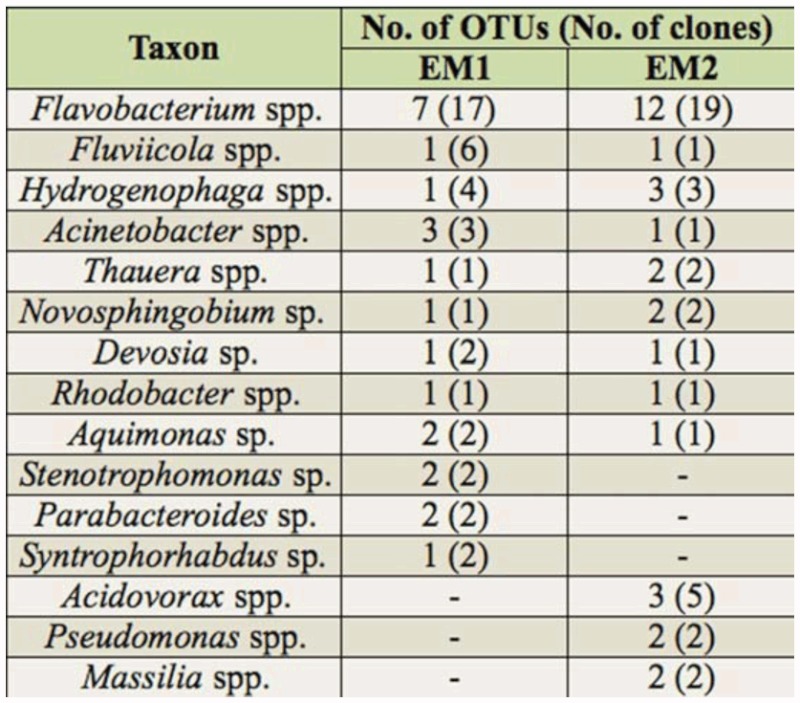
Partial list of bacterial taxa that were identified in at least two clones per one sampled egg mass. Clone libraries were analyzed in the FastGroupll website (http://biome.sdsu.edu/fastgroup/). Ninety-seven percent of sequence similarity was chosen as the Percent Sequence Identity.

### Bacterial isolates resistant to different metals

**Figure 3.**
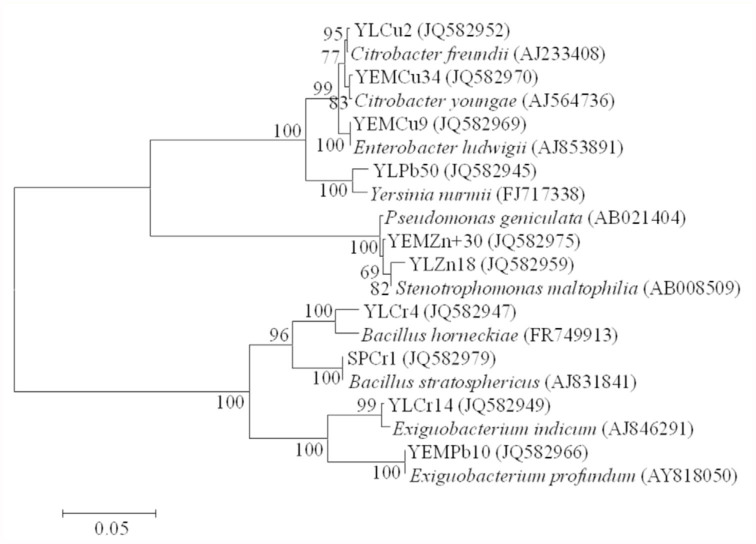
Phylogenetic tree of 16S rRNA gene sequences, representing the bacterial species that were isolated from chironomid egg masses and larvae. These species demonstrated the ability to grow in high metal concentrations. All the isolate names reveal their origin as follows: EM - egg mass, L - larva, Cu - copper resistance, Cr - Chromate resistance, Zn - zinc resistance, Pb - lead resistance. The sequences alignments were performed using CLUSTAL W program and the trees were generated using the neighbor-joining method with Kimura 2 parameter distances in MEGA 4.1 software. Bootstrap values (from 1,000 replicates) greater than 50% are shown at the branch points. The bar indicates 5% sequence divergence. High quality figures are available online.

To further prove that toxicant-resistant bacterial species indeed inhabit chironomid egg masses and larvae, bacteria from egg masses and larvae were isolated on selective media supplemented with the heavy metals K_2_CrO_4_, Pb(NO)_2_, CuSO_4_·5H_2_O and ZnCl_2_. Some of the identified isolates belonged to genera that were found in the clone libraries and the DGGE bands (*Citrobacter, Pseudomonas, Bacillus, Exiguobacterium*) (Figure 3).

### Toxicants concentration in the sampled environment

Heavy metals (lead, chromium, copper, and zinc) were not detected in the Tivon waste stabilization pond from which the egg masses and larvae were sampled.

## Discussion

Analysis and understanding of the environmental microbial communities revealed enormous microbial diversity, expanding our knowledge of their function in a variety of environments (Debroas et al. 2009; Hewson et al. 2009). The phenomenon of specialized bacterial communities associated with a host has been documented in various organisms, such as corals (Rohwer et al. 2002), sponges (Friedrich et al. 2001), and hydra (Fraune and Bosch 2007). Community fingerprinting methods, such as DGGE, allow comparisons of many different samples to answer questions concerning stability of microbial communities.

Here, the bacterial community composition in chironomid egg masses were studied. Bacteria not firmly attached to the egg masses were removed by a rinsing and vortexing procedure. This method had already been proven to be efficient in removing the bacteria that are not residents in this niche (Halpern et al. 2007; Senderovich et al. 2008).

DGGE analysis of bacterial community composition in chironomid egg masses revealed a unique banding pattern in all the samples. This pattern didn't change significantly along the sampling period (Figure 1), demonstrating that specific endogenous bacterial populations are residents in the egg masses and thus can always be found there.

Screening clone libraries provides a comprehensive assessment of the microbial community, but is usually restricted to a limited number of samples due to both financial cost and laboriousness of the approach. No significant differences were found between the clone libraries of the different studied egg masses. The largest OTU, *Flavobacterium* spp., was present in both libraries (Table 1). Clones from different libraries clustered together in phylogenetic trees (Figure 2a,b,c), representing a steady bacterial community composition that can be coupled with chironomid egg masses.

Although chironomids were described in many studies as a stable reservoir of *V. cholerae* (Broza and Halpern 2001; Halpern et al. 2004, 2006, 2007; Broza et al. 2005), none of the clones in the current study were identified as *V.cholerae*. This is not surprising, as *V.cholerae* comprise a minor portion (< 1%) of the bacterial microbiota of egg masses (Halpern et al. 2007) and thus couldn't be detected by the techniques used in the current study. *Aeromonas* spp., which was also described as an inhabitant of chironomids (Senderovich et al. 2008), was found in one clone.

The substrate in freshwater habitats acts as a sink for naturally occurring wastes and different anthropogenic toxic substances (Salomons et al. 1987). Chironomid larvae are known to have a wide range of tolerance for pollution (Winner et al. 1980; Armitage et al. 1995; Thorne and Williams 1997; Halpern et al. 2002), however their protective mechanisms against pollutants are not well understood. The current molecular analyses of the bacterial communities in chironomids (Table 1, Figures 1 and 2) and the previous analysis of culturable bacteria from chironomid egg masses (Halpern et al. 2007) revealed the presence of species known for their ability to degrade toxicants, such as *Shewanella* (Guha et al. 2001), *Acinetobacter* (Lee 1994), *Exiguobacterium* (Okeke 2008), *Leucobacter* (Halpern et al. 2009), and *Pseudomonas* (Williams and Sayers 1994). Furthermore, bacterial species resistant to different heavy metals were isolated from egg masses and larvae (Figure 3). These metal resistant isolates were identified as belonging to the following genera; *Bacillus, Citrobacter, Enterobacter*, Exiguobacterium *Pseudomonas, Stenotrophomonas*, and *Yersinia*. Taxa belonging to the same genera were also identified in the cloning and DGGE analyses, even though the different samples were taken between 2005 and 2008 (see the methods for sampling). The findings of this study demonstrate that endogenous bacterial populations in chironomids are stable. Eller et al. (2007), who investigated the possible sources of methane-derived carbon from chironomid larvae, identified clone libraries from larval tissue and gut content. The majority of the identified clones in their study were related to known detoxifying bacteria and belonged to the following genera: *Bacteroides, Clostridium, Dysgomonas, Hyphomicrobium*, Methylobacillus, *Methylobacter*, Methylocaldum, and *Methylomicrobium*. Bacteroides and *Methylobacillus* were identified in the current study in egg masses clones.

The first instar of chironomid larvae hatch from eggs and feed on the gelatinous matrix of egg masses. This may be the way the larvae become infected with the endogenous bacterial community inhabiting egg masses. The interactions between the host and its microbial community have become the focus of many microbial ecology studies (Nakanishi et al. 1999; Mai-Prochnow et al. 2004; Matsuo et al. 2005; Rao et al. 2005). Insect symbionts may influence host ecology. The bacteria open niches to their insect host that would otherwise be unavailable (Feldhaar 2011).

In conclusion, the stable endogenous bacteria that inhabit egg masses may play a role in protecting their chironomid host from pollutants. Nevertheless, this hypothesis has to be further studied and proven, especially in the larval life stage (Halpern 2011).

## Abbreviations

DGGE,: denaturing gradient gel electrophoresis;
EM,: egg mass
